# Experiences of heat stress and adapting practices among farmworkers in northwest Nicaragua: a qualitative study

**DOI:** 10.1136/bmjopen-2025-115295

**Published:** 2026-03-19

**Authors:** Ana L Pineda Reyes, Andres Jaime, Aurora Aragón, Indiana López-Bonilla, Neil Pearce, Ben Caplin, Marvin González-Quiroz

**Affiliations:** 1 Department of Environmental and Occupational Health, Kate Marmion School of Public Health, The University of Texas at San Antonio, San Antonio, Texas, USA; 2 Office of Research and Partnerships, Kate Marmion School of Public Health, The University of Texas at San Antonio, San Antonio, Texas, USA; 3 Wuku’ Kawoq Maya Health Alliance, Chimaltenango, Guatemala; 4 Department of Medical Statistics, London School of Hygiene and Tropical Medicine, London, UK; 5 Department of Non-Communicable Disease Epidemiology, London School of Hygiene and Tropical Medicine, London, UK; 6 Bladder and Kidney Centre, University College London, London, UK

**Keywords:** Acute renal failure, Chronic renal failure, EPIDEMIOLOGY, Environmental Illness

## Abstract

**ABSTRACT:**

**Objectives:**

Chronic heat stress and recurrent dehydration from strenuous labour in hot environments are recognised drivers of acute kidney injury among agricultural workers in Mesoamerica and may contribute to Chronic Kidney Disease of Unknown Aetiology (CKDu). This study explored how members of a long-term community-based cohort in northwest Nicaragua perceive, experience and adapt to extreme heat, within the broader context of environmental and labour changes.

**Design:**

This qualitative study used focus group discussions with participants from a community-based cohort followed for over a decade and community members. Transcripts were analysed thematically using an interpretative approach, with trustworthiness ensured through peer debriefing, audit trails, triangulation and achievement of thematic saturation.

**Settings:**

Rural agricultural communities in northwest Nicaragua participating in a long-term community-based cohort.

**Participants:**

Participants were purposively sampled from a prospective community-based cohort and community members were invited to participate. Men and women across different age groups were invited. In total, 91 adults aged ≥18 years participated in 11 face-to-face focus groups, each comprising 8–11 men or women.

**Outcomes:**

Themes describing experiences of heat stress, occupational risk and adaptive responses among agricultural workers.

**Results:**

Participants described worsening heat linked to deforestation, unsafe and inadequate water access and unrealistic production targets that prioritised output over health. In response, workers reported adaptive practices including self-paced labour, hydration routines and peer monitoring. Community solidarity and mutual aid emerged as key sources of resilience despite structural constraints.

**Conclusion:**

Heat stress amplifies occupational hazards and exacerbates health inequities among marginalised agricultural workers. Integrating climate adaptation and equity into labour protections—ensuring access to clean water, adequate shade and fair workloads—can strengthen resilience in agricultural communities facing rising heat-related health risks.

**STRENGTHS AND LIMITATIONS OF THIS STUDY**

- The use of an interpretive qualitative design allowed for in-depth exploration of community and workers’ perspectives about heat stress exposure in Nicaragua.
- Sampling ensured representation across age, sex, occupation and communities across northwest Nicaragua.
- Thematic saturation was achieved by the seventh focus group, which strengthens confidence in the completeness of the data.
- Participants were recruited from a longitudinal Chronic Kidney Disease of Unknown Aetiology (CKDu) study or by community leaders, which may introduce selection bias.
- Translating the data after analysis may pose risks of subtle meaning loss during reporting.

## Introduction

 Agricultural workers in hot, labour-intensive environments face increasing occupational and health challenges that compromise their well-being and productivity. In Central America, prolonged heat exposure, limited access to water and productivity-based labour structures have intensified risks of dehydration and kidney damage. These conditions have been linked to the epidemic of Chronic Kidney Disease of Unknown Aetiology (CKDu), a progressive and often asymptomatic illness affecting thousands of young rural labourers in Mesoamerica and South Asia.^1^

In Nicaragua, Chronic Kidney Disease (CKD) ranks as the second leading cause of death after ischaemic heart disease,^2^ with an age-standardised mortality rate of 34.3 deaths per 100 000 population in 2021.^3^ This disease disproportionately affects young, male agricultural workers in their most productive years,^14^,^8^ resulting in premature mortality and profound socioeconomic disruption in rural households.

Evidence increasingly suggests that cumulative kidney injury associated with heat stress, recurrent dehydration and demanding physical work under extreme temperatures leads to decline in kidney function in those at risk from CKDu.^9^ Labour systems emphasising productivity, such as piece-rate wages, and the scarcity of shade, rest and clean water exacerbate these risks.^1^ Beyond its physiological consequences, CKDu entails heavy social and psychological burdens such as loss of income, caregiving strain and restricted access to renal replacement therapies^10 11^ that further exacerbate inequities in Nicaragua.

Despite substantial advances in quantitative research describing CKDu prevalence, risk factors and possible aetiological pathways, far less attention has been paid to the perspectives of those most directly affected. Existing qualitative studies have largely focused on the views of healthcare providers (frontline physicians’ and pharmacists’) or researchers, or on systemic challenges in studying CKDu, rather than on workers’ lived experiences.^12 13^ As a result, critical gaps remain in understanding how workers perceive, interpret heat stress, kidney-related symptoms and how they cope with these risks in their everyday working lives under structurally constrained conditions.^4 11^ To address this gap, this qualitative study explores the lived experiences and adaptive strategies of Nicaraguan agricultural workers regarding heat exposure and kidney health. Specifically, it examines: (i) how workers perceive and experience heat stress and their health risks; (ii) the individual and collective strategies they use to prevent or mitigate heat-related illness; and (iii) the social, cultural and structural factors influencing these practices. By situating CKDu within the lived experiences of rural workers, this research seeks to elucidate the interplay between labour conditions, cultural understandings and adaptive behaviours that shape health protection in contexts of high occupational vulnerability.

## Methods

### Study design

An interpretive qualitative study was used to explore community and agricultural workers’ perceptions, preventive practices and structural barriers related to occupational heat stress and CKDu.

### Reflexivity

The Nicaraguan research team has longstanding experience in CKDu research. The primary moderator [Aurora Aragón (AA)], a physician and occupational health researcher, had prior professional engagement with CKDu but no personal relationship with the specific cohort communities. Her prior knowledge regarding kidney disease and occupational exposures required reflexive awareness to avoid privileging pre-existing explanatory frameworks during interpretation.

The cohort principal investigator [Marvin González-Quiroz (MG-Q)], who has maintained sustained engagement with the communities and communicated laboratory results over several years, did not participate in the groups to minimise potential influence associated with prior relationships or perceived authority.

A third researcher [Indiana López-Bonilla (IL-B)], a PhD statistician, conducted initial participant contact, collected background information and acted as a non-directive observer during sessions, taking field notes and requesting clarification only at the end when needed.

To balance insider contextual knowledge with analytical distance, the first two authors who had no prior involvement in CKDu research in Nicaragua conducted the preliminary coding and thematic development. Findings were subsequently reviewed by the Nicaraguan researchers to ensure contextual accuracy. This collaborative reflexive process supported interpretive depth while minimising the influence of prior assumptions.

### Participants’ characteristics and recruitment

Participants were adults aged 18 or older who were either enrolled in an ongoing 10-year longitudinal CKDu study or identified as community members affected by the disease. We conducted a total of 11 focus group discussions (FGDs), one per participating community between March 5 - 22, 2024. Each FGD included 8 to 11 individuals, for a total of 91 participants (53 females and 38 males). Table 1 outlines the demographic characteristics of the study population. The mean age of the participants was 31±9.47 years old. Employment was defined as currently engaging in paid or unpaid outdoor or heat-exposed work, including agricultural and other manual labour, regardless of contract status (formal, informal or self-employed). Thirty-six participants were engaged in manual labour, including agriculture (n=26) and manufacturing-related work (n=7), at the time of the study. On average, they had been working for 9.8 years in their current occupation, with a range from 1 year to 60 years. Participants were invited through purposive sampling, aiming for balanced representation across age, sex and occupation (eg, sugarcane workers, subsistent agriculture, manufacturing, etc).

**Table 1:** Demographic characteristic reported by participants in the 11 focus groups (n=91)

Characteristics	N (%)
Age range (years)	
20–29	39 (42.9)
30–39	43 (47.3)
40–49	4 (4.4)
≥50	5 (5.4)
Sex	
Female	53 (58.2)
Male	38 (41.8)
Current occupations	
Agricultural	29 (31.9)
Housewife*	33 (36.3)
Manufacturing	7 (7.7)
Other	12 (13.19)
Unemployed†	10 (11.0)

* 12 housewife reported having previously engaged in agricultural work, indicating a dual occupation as both homemakers and agricultural workers.

† Nine participants reported history of agricultural work prior to study enrollment.

Community leaders played an important role in recruitment. They announced the study among study participants and community members affected by CKDu, coordinated suitable venues and extended invitations to interested individuals. Although thematic saturation was achieved after the seventh FGD, all 11 were retained to ensure geographic representation.

### Rigor

To ensure trustworthiness, methodological triangulation was implemented through investigator triangulation, collaborative coding and multidisciplinary team review and regular peer debriefing meetings. An audit trail was maintained throughout data collection and analysis to document coding decision, theme development and analytic reflections. All FGDs were conducted in Spanish. AA moderated each session; IL-B served as a non-directive observer, and MG-Q provided technical and logistical support. These differentiated roles supported systemic data management.

### Procedure

FGDs were initiated using broad, open-ended prompts about participants’ experiences of working in high-heat environments and their perceptions of kidney health. Rather than following a fixed sequence of predetermined questions, discussion evolved conversationally as participants elaborated on their lived experiences. The moderator followed participants’ narratives and used responsive probing to deepen exploration of emerging issues, including work organisation, production pressures, hydration practices, environmental changes and perceived links between heat exposure and kidney health. Although discussions unfolded flexibly, the analysis was guided by recurrent domains that consistently surfaced across sessions. The following analytical questions reflect these domains and structure the thematic analysis presented in this manuscript: (i) How do agricultural workers perceive and experience occupational heat exposure and its implications for their health?; (ii) What preventive and adaptive strategies are currently adopted by agricultural workers, community and workplace levels in response to heat exposure?; (iii) How do work organisation, production demands, and environmental conditions influence workers’ ability to implement heat-prevention practices?; (iv) In what ways do agricultural workers perceive the relationship between heat exposure, dehydration and kidney health? (see online supplemental file 1).

These analytical questions were not posed as fixed or sequential items during the focus groups; rather, they represent the principal thematic domains that emerged through conversational discussion. Two FGDs were performed per day and all sessions started with ice breaker dynamics. Each FGD lasted 60–90 min, was digitally recorded, transcribed verbatim in Microsoft Word, and reviewed by the Nicaraguan research team.

### Patient and public involvement

Participants and/or the public were not involved in the design, conduct, reporting or dissemination plans of this research. However, the findings from the FGDs informed the development of educational material (manual) on heat perception, adaptation practices and CKDu. This material was produced in Spanish and distributed to cohort participants and community members.

### Data analysis

Recorded group sessions were transcribed verbatim by AA and reviewed line-by-line by MG-Q and IL-B. Transcripts were not returned to participants for post-session validation due to time constraints and geographic dispersion of communities; however, clarification and confirmation of meaning were conducted during the FGDs as needed. The transcripts and field notes were independently reviewed and coded by members of the research team using an inductive thematic analysis approach. Ana L Pineda Reyes (ALPR) and Andres Jaime (AJ) independently coded the transcripts line by line to identify recurring patterns. Codes were compared and refined through discussion and then grouped into broader categories and themes. Emerging codes were refined using value coding to capture participants’ beliefs and attitudes.^14^ These codes were then organised into categories aligned with the research questions. To ensure analytic robustness, codes within each domain were examined to identify themes representing shared community experiences. Non-conforming codes were reviewed to preserve critical insights. Through this collaborative process, the coding structure was refined and consolidated into preliminary themes (table 2).

**Table 2:** Coding scheme used to identify themes that reflected the shared experiences of agricultural workers and community members in Nicaragua about heat stress

Parent code	Meaning	Child code	Examples
Heat exposure	Related to higher temperature, longer direct sun exposure that can lead to heat stress and dehydration.	**HTEMP** – High temperatures **AIRPRE** – Air pressure **SHADE** – Less shaded areas **FEWBRK** – Fewer breaks	“*We are also exposed to the sun. In our fields, we don’t leave any trees, and we have no shade to take shelter in, so we expose ourselves too much to the heat.*” (Male, FGD7)
Water consumption	Lack of water intake and increase of soda consumption during working hours which contribute to dehydration.	**DEHY** – Dehydration **ACCESSW** – Limited access to clean water. **SODA** – Soft drink intake	“*The availability of water is not everywhere. When you go to work, there isn’t any*” (Male, FGD2)
Production and work environment	Connected to actions taken by employers and workers to maximise production and earn higher piece-rate wages. Such practices often include setting excessive work quotas or continuing to work beyond safe limits to increase pay.	**WDEMA** – Imposed excessive work goals/quotas **LHOURS** – Long work hours **FEWBRK** – Fewer breaks **SHADE** – Less shaded areas	“*Sometimes there are companies [that] don’t give you any time. There’s no time even to eat. Sometimes they just say 15 minutes.*” (Male, FGD1)
Increase hydration	Pertaining to high intake of water or ‘frescos naturales’ to stay hydrated and prevent heat stress while working.	**HYDR** – Hydration **PLANTB** – Plant-based remedies **HOMEM** – Home-made remedies to prevent dehydration, ie, frescos naturales and oral rehydration solution	“*That they [agricultural workers]) drink plenty of water, about 6 liters, depending on how much the person works*” (Male, FGD10)
Responsible working habits	Relating to workers’ and employers’-initiated measures in their work environment aimed to protect their physical health, to prevent heat stress.	**REST** – Work breaks **WTRAIN** – Work trainings related to operation and field work **MSHADE** – Access to shaded areas during work	“*Don't push your body to the limit, work but at a normal pace, you know what I mean.*” (Male, FGD8)
Self and community health	Connected to worker-initiated measures aimed to protect their, and the communities’ health and prevent CKDu. These practices can range from body awareness, healthier dietary choices and community transfer of knowledge.	**HFOOD** – Healthy food intake **BODYQ** – Learning to listen to our own bodies and respond appropriately as the basis for self-care **WTRAIN** – Work trainings related to operation and field work	“*We can raise awareness among more people so that we can properly take care of ourselves, through nutrition, health in our workplace and develop more consciousness toward others, because as employers or workers, we can harm people.*” (Male, FGD1)

CKDu, Chronic Kidney Disease of Unknown Aetiology; FGD, focus group discussion.

The final analysis was reviewed and discussed with the broader study team (MG-Q and AA) to validate interpretations and resolve any discrepancies. Discussion regarding further refinement of the themes and codes was conducted among all team members. Findings are presented using descriptive summaries and illustrative participant quotations to enhance transparency and support the credibility of the results. Data saturation was assessed continuously and reached when no new codes or themes emerged. Triangulation was strengthened through the involvement of a multidisciplinary research team with expertise in qualitative research, occupational health, epidemiology, nephrology and research methods. All analyses were conducted in Spanish using MAXQDA 2024 software (VERBI Software, 2024), with selected quotes later translated into English for reporting.

## Results

The analysis identified multiple parent and child codes reflecting consistent statements about high temperatures, hydration, challenges within the labour system and preventive practices to reduce heat stress risks both during work hours and at home.

Five principal themes were identified: (i) worsening heat conditions; (ii) production quota as a central factor of heat stress; (iii) staying hydrated to combat heat; (iv) self-advocacy for health working practices; and (v) self and community well-being practices. These themes capture agricultural workers’ perceptions of heat stress, and the preventive measures they employ to protect their health.

### Worsening heat conditions

Participants’ perceptions of heat stress and its prevention were closely tied to worsening environmental and occupational conditions, particularly prolonged exposure to extreme temperatures. Workers described a daily reality of labouring under the sun for extended hours, often without adequate hydration or opportunities to rest.

Usually when we’re working in the fields, it’s during the hottest hours*.* (Male, FGD1)

That really is hard work, under the midday sun, that is really is tough work. You sweat a lot and everything*.* (Male, FGD8)

Environmental degradation, particularly deforestation, was perceived to exacerbate these conditions by eliminating natural sources of shade.

There’s a lot of deforestation. (Male, FGD7)

In our fields, we don’t leave any trees, and we have no shade to take shelter in, so we expose ourselves too much to the heat. (Male, FGD7)

Participants linked heat exposure to a range of physical strain and health problems. One participant described the bodily toll as a kind of ‘*internal poisoning*’.

Above all, it’s like you’re poisoning your body from all the sun rays that hit you. (Male, FGD7)

Others associated prolonged sun exposure with urinary symptoms such as strangury, locally known as *chistata*.

Sometimes when I get overexposed to the sun I suffer from chistata. That is where it all comes from. (Male, FGD5)

### Barriers to hydration

Participants reported significant barriers to hydration, as clean drinking water was often unavailable.

The availability of water is not everywhere. When you go to work, there isn’t any. (Male, FGD2)

Even when water was accessible, its quality was questioned due to potential contamination by agricultural chemicals used for their crops.

Also, the water is polluted*.* (Female, FGD6)

Well, the chemicals they spray on the sugarcane seep into the water*.* (Male, FGD2)

The amount of water decreases, and the little water that we do get to drink is mixed with those chemicals*.* (Male, FGD1)

The lack of potable water led some to instead consume soda or other sugary drinks, further contributing to dehydration.

There are people who say, ‘I’m going to have a soda instead to satisfy my thirst’ and that is bad*.* (Female, FGD4)

Sometimes we overdo it with the drinks we take, like sodas, we don’t even drink natural juices. (Female, FGD1)

Workers recognised that inadequate hydration, combined with prolonged exposure to high temperatures, was frequently linked to early signs of physical strain and kidney discomfort.

We sweat so much, and sometimes we don’t hydrate properly like we should. (Male, FGD7)

Pressures [referring to the temperature] here in the western region are extremely high. And not drinking water causes weakness in our kidneys. (Male, FGD9)

### Production quota as a central factor of heat stress

Workers consistently described sacrificing basic needs such as hydration and rest to meet production quotas—whether imposed by employers or internalised as self-expectations—to maintain job security and income.

Time pressures created by employers left many workers with little opportunity to rest, eat or hydrate adequately during the workday.

Sometimes there are companies [that] don’t give you any time. There’s no time even to eat. Sometimes they just say 15 minutes. (Female, FGD1)

Under these restrictions, combined with production demand, workers were led to adopt extreme behaviours, including delaying water intake despite high heat and physical exertion.

There are people who get obsessed, and to meet a quota, because we’re given production targets, many don’t get up to drink water whenever they’re thirsty. They only go when they remember or when they truly can’t stand the thirst anymore. (Female, FGD1)

We barely drink any liquids, we leave it for the end, so we don’t fall behind. (Male, FGD9)

This mindset extended to informal norms in the field, as several workers described that they pushed beyond their physical limits to keep pace with others. This competitive environment also prompted hydration delays, heightening the pressure to maintain a rapid work pace.

Someone who works in the fields, in the sugarcane, in order not to fall behind, they stop drinking water*.* (Female. FGD9)

I wouldn’t drink water until I had completed 100 lines, they call them 100 varas, so I could keep moving forward*.* (Male, FGD7)

When there is someone better than you at work, and you are both coworkers, you have to push yourself and give it you all, so your coworker doesn’t outdo you. (Male, FGD4)

The consequences of this productivity-over-health mindset were clearly recognised by the workers themselves.

Just to earn a little more, you end up ruining your body, because you’re pushing past your limits. You’re overworking your body, dehydrating your kidneys. (Male, FGD7)

Participants emphasised that production pressures, combined with an internalised willingness to sacrifice their bodies to meet work demands, exacerbate barriers to staying hydrated and taking adequate work breaks, putting them at greater risk of heat-related illness.

### Staying hydrated to combat heat

The interviews also identified the proactive strategies that workers use to stay hydrated and protect their health while working in extreme heat exposure. These preventive practices are rooted in both personal experience and cultural knowledge.

Many participants expressed a strong sense of personal responsibility over their own health, emphasising the importance of water intake as a preventive measure.

We are the owners of our own bodies, we are the ones who need to know what kind of things we should put into our bodies, not drinking [alcohol], drink plenty of water*.* (Male, FGD7)

The more exhausted [you are] the more water you need to drink. (Male, FGD10)

Participants also described specific strategies to make water consumption more appealing and effective during long workdays. Because drinking warm water in extreme heat was viewed as both unpleasant and less effective, workers adapted practical methods to keep their water cold and increase hydration. A common approach involves wrapping jugs in damp cloth or sack material.

Everyone carried their jug wrapped up. (Male, FGD7)

Moreover, participants emphasised that the timing of hydration was critical for preventing heat-related illness. Many noted that maintaining consistent hydration—rather than drinking only when thirsty—was essential for protecting kidney health and preventing heat stroke.

If you drink water constantly, at the right time, there’s no reason you should get it [referring to the CKD], and your kidneys is healthy*.* (Male, FGD8)

I recommend that we drink enough water so that our Creatinine levels do not rise. (Male, FGD4)

In addition to water, some participants relied on oral rehydration solutions, commonly referred to as *Suero*, as well as simple home remedies to replace fluids and electrolytes lost through sweating.

Drink a lot of water. Add a teaspoon of sugar to a glass of water. (Female, FGD9)

Thus, despite significant structural and occupational challenges, workers actively engage in proactive practices to maintain hydration.

### Self-advocacy for healthy working practices

The interviews revealed that many workers have developed their own responsible working habits to protect their health and manage heat stress in the absence of formal protection. These worker-initiated strategies, grounded in practical experience, include pacing their labour, taking breaks in shaded areas and adjusting work schedules to avoid peak heat hours. Such practices reflect an increasing awareness of the health risks associated with prolonged heat exposure and the importance of self-regulation in the field.

Participants consistently emphasised the need to listen to their bodies and avoid pushing beyond physical limits during the workday. As one worker reflected, maintaining a manageable pace was essential for sustaining both productivity and well-being.

Don't push your body to the limit, work but at a normal pace, you know what I mean. (Male, FGD7)

Another worker described the importance of being aware of one’s physical condition and prioritising hydration and rest as essential components of sustainable fieldwork.

Field work is very hard, but you also must be aware that [even when] working in the fields, you need to take care of yourself. You can’t neglect drinking water or natural drinks, take time in the shade. I do that. (Male, FGD1)

Avoiding the hottest hours of the day also emerged as a common protective strategy:

I always protect myself and avoid working during the hottest hours of the day*.* (Male FGD1)

Access to shade and intentional rest periods were frequently mentioned as vital coping mechanisms, especially when working under direct sun exposure. Many workers described intentionally and deliberately scheduling time to cool down, rest and recover.

Take your time in the shade, because I do that too. Give your body time in the shade so it doesn’t wear out. (Female, FGD1)

Four hours of work, then we can go out and rest for a while. (Male, FGD8)

Midday breaks were also described as an essential routine, allowing workers to regulate body temperature and avoid heat exhaustion:

We always take half an hour, or even an hour, at midday. We rest a little in the shade. (Male, FGD8)

Despite often lacking formal occupational protections, workers have developed practical and self-directed methods to reduce heat-related risks.

While these individual strategies demonstrate self-responsibility and adaptation, participants also described how protecting one’s health was inseparable from caring for others. This transition from individual to collective concern introduces the next theme on self and community well-being.

### Self and community well-being practices

Participants discussed both personal and collective strategies for mitigating the effects of heat stress. They conveyed a strong sense of personal responsibility over their health, acknowledging that prevention begins with understanding one’s own body and needs.

We are the owners of our own bodies; we are the ones who need to know what kind of things we should put into our bodies*.* (Male, FGD7)

This body awareness translates into deliberate decisions about nutrition and rest to preserve physical resilience and prevent illness.

As one participant stated, They’re mostly focused on working, but they also have to “echarle al cuerpo” (feed their bodies), eat because if a person does not eat well, they will gradually weaken and that harms them*.* (Female, FGD1)

Participants highlighted the need to recognise the body’s signals and adjust their behaviour accordingly. Over time, workers reported learning to adapt to environmental demands while understanding their own physical limits.

The body adapts, the body knows it’s going to get those 15 minutes of rest, and it starts asking for it. (Male, FGD8)

The body has a limit, maybe it’s already saying ‘no more,’ but we push it anyway. We know the work will still be there, and in the end, we’re the ones who die. (Female, FGD9)

Alongside self-care, participants highlighted the importance of community-based health promotion. Many expressed a desire to raise awareness among peers about preventive behaviours to collectively reduce illness. These preventive behaviours were often shared informally.

It’s through communication person to person; one person tells another. (Female, FGD6)

People need to truly understand what they need to do to avoid getting sicker. (Female, FGD9)

Some framed this knowledge-sharing as both a moral duty and a way to protect loved ones.

We can raise awareness among more people so that we can properly take care of ourselves, through nutrition, health in our workplace and develop more consciousness toward others, because as employers or workers, we can harm people. (Male, FGD1)

To take care of ourselves so that, in a way, we also take care of our family*.* (Male, FGD4)

These statements reflect how knowledge functions as both a personal and collective tool for protecting agricultural workers’ health and well-being. As a male participant noted, “*we need to take action*” (FGD7), emphasising the role of fostering preventive habits within the community to strengthen individual resilience and promote collective well-being.

## Discussion

This qualitative study explored agricultural workers’ perceptions of heat stress risks and preventive practices in Nicaragua, a country severely affected by CKDu. By centring workers’ own narratives, this study addresses a critical gap in the CKDu literature, which has been dominated by epidemiological, clinical and environmental exposure studies, with limited attention to how heat stress and kidney risk are understood, experienced and managed in daily working life. Our findings reveal how worsening environmental conditions, labour demands and limited access to clean water collectively intensify heat-related risks. Worker’s narratives illustrate how climate change intersects with local ecological degradation, particularly deforestation, to heighten exposure and reduce opportunities for cooling.

The absence of shade and persistent high temperatures contribute to chronic dehydration and physiological strain, consistent with evidence linking recurrent heat stress and dehydration to CKDu.^1 9^ Workers emphasised how deforestation worsens heat exposure by eliminating natural shade, increasing ambient temperatures that contribute to increasing core body temperature and dehydration and thereby intensifying physiological strain. The absence of shaded, rest areas in agricultural fields forces workers to rest under severe heat conditions, which fails to cool their bodies effectively. While previous studies have documented the effects of global warming on occupational health and worker’s productivity.^15^,^18^ This study adds qualitative insights into how local ecological changes are perceived by workers and how these changes directly shape their capacity to rest, cool down and protect their health during work.

Hydration emerged as both a prevention strategy and a major occupational challenge. Workers are constantly sweating, which contributes to significant body weight loss during the harvesting season, while adequate hydration can reduce creatinine increase and protect against Acute Kidney Injury (AKI).^19^ However, limited access to clean drinking water remains a barrier. Participants expressed concerns about water scarcity and contamination, which aligns with previous research linking exposure to environmental toxins such as glyphosate and heavy metal exposure to CKDu.^20^,^22^ Despite these risks, workers often drink large volumes of water to protect themselves from heat. In hypothetical scenarios where water is contaminated, this behaviour could increase the risk of kidney damage, whereas insufficient water intake may also be harmful due to reduced kidney perfusion.^7^ In response, some substitute water with soft drinks or other beverages, potentially introducing additional health risks.

The substitution of water with sugary beverages reflects structural determinants of health—economic pressure, lack of potable water and workplace expectations—that shape maladaptive coping behaviours and may worsen renal risk.^23^ Furthermore, some companies have introduced oral hydration solutions containing high levels of glucose; however, these products may be more harmful than protective.^24 25^ By documenting how workers interpret and use these interventions, this study highlights the gap between formal prevention guidelines and lived practice. Additionally, production quotas emerged as a key determinant of dehydration and heat stress, as workers often deprioritised rest and hydration to meet productivity targets. These pressures push workers beyond safe physical limits, compromising kidney health and encouraging maladaptive coping behaviours.

Community knowledge and informal support networks played an important role in shaping preventive strategies. Health protection was framed as a collective moral responsibility, reflecting how decision-making is embedded in social relationships. This aligns with evidence that CKDu has deep social and economic impacts at the household and communities’ level, and that community networks are central to coping and resilience.^8 11^ Future interventions should therefore leverage existing social networks and peer-to-peer education to strengthen the adoption of preventive behaviours among the community.

This study has several strengths. First, by employing a qualitative approach focused on lived experience and everyday practice—an approach that remains scarce in the CKDu literature—it provides original insights into how heat stress and kidney risk are understood and managed by workers themselves. Second, participants represented diverse occupations, including sugarcane work, subsistence agriculture, construction, manufacturing and other labour sectors, providing a comprehensive view of heat stress exposure across employment types and revealing prevention strategies implemented by workers, companies and communities to mitigate the impact of heat stress on kidney health. Third, participants from across the community allowed inclusion of individuals with normal kidney function and those affected by CKDu. Fourth, the study captured participants’ perceptions and practices related to heat stress, highlighting urgent intervention needs linked to environmental degradation, structural inequities and poor labour conditions. Finally, thematic saturation was achieved after seven focus groups, yet eleven were conducted to ensure representation across municipalities differing in road access, healthcare availability and access to safe drinking water and heat-stress training.

Several limitations should be noted. First, the findings are context-specific to rural communities in Nicaragua and may not be generalisable to other affected regions, as populations differ in their occupation practices, environmental exposures and sociocultural contexts. Second, the study did not assess the clinical effectiveness of the preventive strategies reported by workers in reducing heat stress or mitigating kidney damage. Third, as a qualitative study, the findings are based on self-reported perceptions and experiences, which may be influenced by recall bias or social desirability.

## Conclusion

This study provides critical qualitative evidence that complements epidemiological findings on CKDu, showing how agricultural workers’ perceptions of heat stress are shaped by structural challenges and ecological changes. These perceptions influence their preventive behaviours and coping strategies within both individual and collective frameworks. In Nicaragua, rising temperatures and systemic barriers further heighten CKDu risk, and although workers demonstrate resilience and self-efficacy, sustainable progress demands structural reform. Together, the findings underscore the need for comprehensive, multi-level interventions that address labour conditions, access to safe water and environmental protection, while simultaneously empowering communities through education, advocacy and collective action. Such an integrated approach—combining individual initiative with systemic support—is essential to reducing CKDu risk and promoting long-term health and well-being in affected communities.

## Supplementary material

10.1136/bmjopen-2025-115295 online supplemental file 1

## Data Availability

Data are available upon reasonable request.
