# Supplementary material for: Experiences of heat stress and adapting practices among farmworkers in northwest Nicaragua: a qualitative study
Source: BMJ Open. 2026 Mar 19;16(3):e115295. doi: 10.1136/bmjopen-2025-115295 (PMC13007072; doi:10.1136/bmjopen-2025-115295)
Supplement: online supplemental file 1 [file bmjopen-16-3-s001.docx]

**GROUP DISCUSSION GUIDE**

The focus groups were initiated using broad, open-ended prompts about participants’ experiences of working in high-heat environments and their perceptions of kidney health. Rather than following a rigid sequence of predetermined questions, discussions unfolded conversationally, allowing participants to describe their lived experiences in their own terms. As themes emerged, the moderator followed relevant threads and used probing questions to deepen exploration of perceived risk factors such as heat exposure, production pressures, hydration barriers, environmental change, and adaptive practices.

The guiding prompts used during the discussions are provided below to illustrate the conversational structure of the sessions:

| Subject | Spanish | English |
| --- | --- | --- |
| Experiencing Heat at work | ¿Cómo viven ustedes el calor durante la jornada de trabajo? | How do you experience the heat during your workday? |
| Perceived Environmental Change | ¿Sienten que el calor ha cambiado con el tiempo? ¿Qué ha cambiado? | Do you feel that the heat has changed over time? What has changed? |
| Perceived Risk Factors | ¿Qué cosas creen ustedes que pueden estar afectando la salud de los trabajadores? | What factors do you think may be affecting workers’ health? |
| Work Organization and Production Pressure | ¿De qué manera las metas de producción influyen en el tiempo que tienen para descansar o hidratarse? | How do production targets influence the time you have to rest or hydrate? |
| Access to Water and Hydration | Cuando están trabajando, ¿siempre tienen acceso a agua limpia? ¿Qué hacen cuando no hay suficiente agua? | When you are working, do you always have access to clean water? What do you do when there is not enough water? |
| Heat, Dehydration, and Kidney Health | ¿Cómo creen que el calor y la deshidratación afectan los riñones? | How do you think heat and dehydration affect the kidneys? |
| Adaptive Practices | ¿Qué hacen ustedes para protegerse del calor durante el trabajo? | What do you do to protect yourselves from the heat while working? |
| Collective Strategies | ¿Qué recomendaciones se dan entre ustedes para evitar enfermarse por el calor? | What recommendations do you share among yourselves to avoid becoming ill from the heat? |
