# Supplementary material for: Experiences of heat stress and adapting practices among farmworkers in northwest Nicaragua: a qualitative study
Source: BMJ Open. 2026 Mar 19;16(3):e115295. doi: 10.1136/bmjopen-2025-115295 (PMC13007072; doi:10.1136/bmjopen-2025-115295)
Supplement: Supplementary file 2 [file bmjopen-2025-115295.reviewer_comments.pdf]

PEER REVIEW HISTORY

BMJ Open publishes all reviews undertaken for accepted manuscripts. Reviewers are asked to complete a checklist review form and are provided with free text boxes to elaborate on their assessment. These free text comments are reproduced below.

ARTICLE DETAILS

Title (Provisional)

Experiences of Heat Stress and Adapting Practices among Farmworkers in Northwest Nicaragua: A Qualitative Study

Authors

Pineda Reyes, Ana L; Jaime, Andres; Aragón, Aurora; Lopez-Bonilla, Indiana; Pearce, Neil; Caplin, Ben; Gonzalez-Quiroz, Marvin

---

VERSION 1 - REVIEW

**Reviewer**	**1**
**Name**	**Maric, Nada**
**Affiliation**	**University of Banja Luka**
**Date**	**30-Jan-2026**
**COI**	**None**

Dear author,

This manuscript addresses a highly relevant and timely public health issue at the intersection of occupational heat exposure, climate change, and Chronic Kidney Disease of Unknown Etiology. The use of a qualitative approach embedded within a long-standing community-based cohort represents a significant strength and provides valuable insight.

The introduction effectively summarizes the epidemiological burden of CKDu in Nicaragua, but could be streamlined to more explicitly highlight the qualitative research gap this study addresses.

The study is methodologically sound and clearly described.

However, greater consistency in the description of the analytical approach is recommended, as the manuscript alternates between “interpretive qualitative design,” “qualitative content analysis,” and “thematic analysis.” Adopting and consistently applying one term.

The results compellingly demonstrate both structural vulnerabilities and adaptive strategies among agricultural workers.

Overall, this is a strong and policy-relevant qualitative study that would be suitable for publication in BMJ Open following minor revisions to improve clarity, consistency, and framing.

**Reviewer**	**2**
**Name**	**Nerbass, Fabiana**
**Affiliation**	**Pró-Rim Foundation**
**Date**	**09-Feb-2026**
**COI**	**None**

The authors are to be congratulated for exploring this topic using a qualitative approach that prioritizes listening to the community's perspectives. Given that this methodological approach has been scarcely used in the existing literature on this subject, the authors are encouraged to explicitly highlight this gap in the Discussion, as it reinforces the relevance, originality, and contribution of the present study.

However, clarification is needed regarding the description of participants' employment status. The manuscript states that 81 participants were employed, while Table 1 indicates that 33 participants were housewives. Please clarify how employment was defined in this study and reconcile this apparent inconsistency to improve clarity and transparency.

VERSION 1 - AUTHOR RESPONSE

Referee	***Comments from reviewer***	***Response to comments***
Reviewer 1	*The use of a qualitative approach embedded within a long-standing community-based cohort represents a significant strength and provides valuable insight*	*We thank the reviewer for the positive evaluation of our manuscript*
	The introduction effectively summarizes the epidemiological burden of CKDu in Nicaragua, but could be streamlined to more explicitly highlight the qualitative research gap this study addresses	*Thank you for your suggestion, we have included a paragraph that highlights the gap:*<br>*"Despite substantial advances in quantitative research describing CKDu prevalence, risk factors and possible etiological pathways, far less attention has been paid to the perspectives of those most directly affected. Existing qualitative studies have largely focused on the views of healthcare providers (frontline physicians' and pharmacists') or researchers, or on systemic challenges in studying CKDu, rather than on workers' lived experiences.(12, 13) As a result, critical gaps remain in understanding how workers perceive, interpret heat stress, kidney related-symptoms and how they cope with these risks in their everyday working lives under structurally constrained condition"*

	However, greater consistency in the description of the analytical approach is recommended, as the manuscript alternates between "interpretive qualitative design," "qualitative content analysis," and "thematic analysis." Adopting and consistently applying one term.<br>*The results compellingly demonstrate both structural vulnerabilities and adaptive strategies among agricultural workers.*	*We thank the reviewer for pointing out the inconsistency in our analytical approach. To address this, we have revised the section for consistency and standardized the terminology. Please refer to the updated text below:*<br><br>*Recorded group sessions were transcribed verbatim by AA and reviewed line-by-line by MGQ and ILB. Transcripts were not returned to participants for post-session validation due to time constraints and geographic dispersion of communities; however, clarification and confirmation of meaning were conducted during the focus group discussions as needed. The transcripts and field notes were independently reviewed and coded by members of the research team using an inductive thematic analysis approach. Ana Pineda Reyes (APR) and Andres Jaime (AJ) independently coded the transcripts line by line to identify recurring patterns. Codes were compared, and refined through discussion, then grouped into broader categories and themes. Emerging codes were refined using value coding to capture participants' beliefs and attitudes(15). These codes were then organized into categories aligned with the research questions. To ensure analytic robustness, codes within each domain were examined to identify themes representing shared community experiences. Nonconforming codes were reviewed to preserve critical insights. Through this collaborative process, the coding structure was refined and consolidated into preliminary themes (Table 2).*<br><br>*The final analysis was reviewed and discussed with the broader study team (MGQ and AA) to validate interpretations and resolve any discrepancies. Discussion regarding further refinement of the themes and codes was conducted among all team members. Findings are presented using descriptive summaries and illustrative participant quotations to enhance transparency and support the credibility of the results. Data saturation was assessed continuously and reached when no new codes or themes emerged. Triangulation was strengthened through the involvement of a multidisciplinary research team with expertise in qualitative research, occupational health, epidemiology, nephrology and research methods. All analyses were conducted in Spanish using MAXQDA 2024 software (VERBI Software, 2024), with selected quotes later translated into English for reporting.*
Reviewer 2	*The authors are to be congratulated for exploring this topic using a qualitative approach that prioritizes listening to the community's perspectives.*	*We acknowledge and appreciate the reviewer's positive evaluation.*
	Given that this methodological approach has been scarcely used in the existing literature on this subject, the authors are	*We thank the reviewer for the helpful suggestion to emphasize gaps. Accordingly, we have revised the first*

	encouraged to explicitly highlight this gap in the Discussion, as it reinforces the relevance, originality, and contribution of the present study.	*paragraph of the Discussion and the Limitations section. The same concern in the Introduction has been carefully addressed. The updated text is provided below:*<br><br>*This qualitative study explored agricultural workers' perceptions of heat stress risks and preventive practices in Nicaragua, a country severely affected by CKDu. By centring workers' own narratives, this study addresses a critical gap in the CKDu literature, which has been dominated by epidemiological, clinical and environmental exposure studies, with limited attention to how heat stress and kidney risk are understood, experienced and managed in daily working life. Our findings reveal how worsening environmental conditions, labour demands, and limited access to clean water collectedly intensify heat-related risks. Worker's narratives illustrate how climate change intersects with local ecological degradation, particularly deforestation, to heighten exposure and reduce opportunities for cooling.*<br><br>*This study has several strengths. First, by employing a qualitative approach focused on lived experience and everyday practice- an approach that remains scarce in the CKDu literature- it provides original insights into how heat stress and kidney risk are understood and managed by workers themselves. Second, participants represented diverse occupations, including sugarcane work, subsistence agriculture, construction, manufacturing and other labour sectors, providing a comprehensive view of heat stress exposure across employment types and revealing prevention strategies implemented by workers, companies, and communities to mitigate the impact of heat stress on kidney health. Third, participants from across the community allowed inclusion of individuals with normal kidney function and those affected by CKDu. Fourth, the study captured participants' perceptions and practices related to heat stress, highlighting urgent intervention needs linked to environmental degradation, structural inequities, and poor labour conditions. Finally, thematic saturation was achieved after seven focus groups, yet eleven were conducted to ensure representation across municipalities differing in road access, healthcare availability, and access to safe drinking water and heat-stress training.*
	However, clarification is needed regarding the description of participants' employment status. The manuscript states that 81 participants were employed, while Table 1 indicates that 33 participants were housewives. Reconcile this apparent inconsistency to improve clarity and transparency.	*We appreciate the reviewer's comment and apologize for the confusion regarding the classification of agricultural workers and manual labor. The error occurred during the summation process, as all participants were mistakenly categorized as agricultural and manual workers, with the exception of the unemployed group.*

		In the revised text, we have clarified that 26 participants were agricultural workers and that 7 were employed in the manufacturing sector, which also involves manual labor. It is important to note that of the 10 participants who were unemployed at the time of the study, nine had a history of agricultural work in the months prior to the focus groups.<br><br>*Similarly, within the group of housewives,* ***12*** *participants reported having previously engaged in agricultural work, indicating a dual occupation as both homemakers and agricultural workers*
	Please clarify how employment was defined in this study.	*Thank you for requesting the employment definition used in our study. Please see the definition below:*<br><br>*“Employment was defined as currently engagement in paid or unpaid outdoor or heat exposed work, including agricultural and other manual labour, regardless of contract status (formal, informal or self-employed)”*
